# Acceptance of routine or case-based inquiry for intimate partner violence: a mixed method study

**DOI:** 10.1186/1471-2393-13-77

**Published:** 2013-03-26

**Authors:** Heidi Stöckl, Linda Hertlein, Isabelle Himsl, Nina Ditsch, Carolin Blume, Uwe Hasbargen, Klaus Friese, Doris Stöckl

**Affiliations:** 1 Social and Mathematical Epidemiology Group, London School of Hygiene and Tropical Medicine, 15-17 Tavistock Place, London, WC1H 9SH, UK; 2 Department of Obstetrics and Gynaecology, Campus Grosshadern, Ludwig-Maximilians-University, Marchioninistr.15, Munich, 81377, Germany; 3 Institute of Epidemiology II, Helmholtz Zentrum München, German Research Center for Environmental Health, Ingolstädter Landstr. 1, Neuherberg, 85764, Germany

**Keywords:** Violence, Routine inquiry, Case-based inquiry, Antenatal care, Germany, Mixed methods

## Abstract

**Background:**

The prevalence and detrimental health effects of intimate partner violence have resulted in international discussions and recommendations that health care professionals should screen women for intimate partner violence during general and antenatal health care visits. Due to the lack of discussion on routine or case-based inquiry for intimate partner violence during antenatal care in Germany, this study seeks to explore its acceptability among pregnant German women.

**Methods:**

A mixed methods approach was used, utilizing a self-administered survey on the acceptability of routine or case-based inquiry for intimate partner violence in a university hospital’s maternity ward in Munich and in-depth interviews with seven women who experienced violence during pregnancy.

**Results:**

Of the 401 women who participated in the survey, 92 percent were in favor of routine or case-based inquiry for intimate partner violence during antenatal care. Acceptance of routine or case-based inquiry for intimate partner violence during antenatal care was significantly associated with women’s experiences of child sexual abuse, being young, less educated, single or divorced and smoking during pregnancy. Open-ended survey questions and in-depth interviews stressed adequate training for screening, sufficient time and provision of referral information as important conditions for routine or case-based inquiry for intimate partner violence.

**Conclusions:**

Women in this study showed an overwhelming support for routine or case-based screening for intimate partner violence in antenatal care in Germany. Until adequate training is in place to allow providers to inquire for intimate partner violence in a professional manner, this study recommends that health care providers are made aware of the prevalence and health consequences of violence during pregnancy.

## Background

Violence against women, which is often committed by an intimate partner, has been recognized as an important public health and human rights issue [1]. In recent years, attention has increasingly focused on intimate partner violence during pregnancy due to its prevalence, adverse health consequences and intervention potential. International studies on intimate partner violence during pregnancy show a prevalence of one to 26 percent for physical violence [2], with most studies conducted in Europe reporting a prevalence of one to five percent [3,4]. A representative household survey in Germany found lifetime prevalence rates of physical or sexual intimate partner violence of 25 percent [5]. A maternity ward survey in Munich, Germany estimated a prevalence of intimate partner violence during pregnancy of two percent [6]. Health consequences of intimate partner violence are vast [7]. During pregnancy, intimate partner violence can impact the wellbeing of the mother and child, leading to fetal growth retardation, low birth weight, premature labour, anaemia and increased levels of depression [6,8,9]. These detrimental health effects have resulted in international discussions and recommendations that health care professionals should screen women for intimate partner violence during general health care visits [10,11]. There are two approaches to screening for intimate partner violence, either universal or routine screening of all women regardless of presumed risk, which will be referred to as routine inquiry in the following or case-based screening or clinical inquiry of those women who are suspected to be most at risk or who have known risk factors for intimate partner violence, which will be referred to as case-based inquiry [12-14].

Demands for routine or case-based inquiry during antenatal care are even higher since pregnancy has been identified as a potential window of opportunity for identifying and assisting women who experience intimate partner violence [15]. Antenatal care is often the only point of contact of women with the health care setting and its nature to provide health services and support through the duration of a pregnancy allows healthcare providers to build up the necessary rapport to address intimate partner violence effectively. In addition to that, it has been found that intimate partner violence increases during pregnancy and that women who experience intimate partner violence during pregnancy are also more likely to experience severe intimate partner violence [2,16,17]. For example, a recent systematic review established that pregnancy is a risk factor for intimate partner homicide and attempted suicide [16].

Previous studies on routine inquiry for intimate partner violence had mixed findings on whether women’s prior experiences of abuse affected their acceptability of routine inquiry [18-22]. Differences also emerged in regard to socio-economic characteristics, with younger women feeling more embarrassed by routine or case-based inquiry than older women [19,20] and women with a high school degree or higher income being more likely to oppose it [20,23]. As it has been reported before, health care providers are often hesitant to inquire about intimate partner violence. They are concerned that they offend or alienate women who might already feel stigmatized due to their experiences of abuse, mental health issues or substance abuse. For example, prior research has shown that women who abuse substances may not seek prenatal care for fear of being judged or losing child custody [24].

In Germany, screening for intimate partner violence has only been examined by few studies. A study by Hellbernd et al. found that 79 percent of women attending an emergency department would find it acceptable if doctors ask about intimate partner violence [25]. Authors by the same study recommended several screening instruments and support for screening [14]. Apart from that, no empirical work has been conducted in Germany on whether women are more accepting of routine or case-based inquiry in antenatal versus general care, their perceptions on it or the conditions under which they would like doctors to routinely inquire for intimate partner violence. The aim of this study therefore is to provide first evidence to close this gap in knowledge. We hypothesize that women will be more open to routine or case-based inquiry in antenatal care than in general care and that women’s socio-demographic characteristics, such as age and education as well as adverse behaviours during pregnancy such as drinking or smoking will influence this acceptance.

## Methods

This study combines results of a quantitative, cross-section survey, with closed and open-ended questions of women attending a maternity ward and findings from qualitative in-depth interviews with survey participants, who experienced intimate partner violence.

### Survey design and participants

Study details have been described in detail elsewhere [6]. In brief, the survey was conducted among all adult women, who spoke sufficient German and who were patients in a maternity ward in a university hospital in Munich from 2007 until 2008. Doctors approached all eligible women one to seven days after delivery if they could talk to them in private. Doctors informed the women about the study and its voluntary and confidential nature. They stressed safety precautions when handing over the questionnaire and informed consent sheet. The women could fill in the questionnaire in their own time and return it in the enclosed envelope. Each participant also gave written informed consent to link the questionnaire information to data from their medical file. For anonymity purposes, only one author (DS) could link the medical file data to the survey data. The end of the questionnaire included information on violence services in and around Munich and the free 24 hours psychological support or pastoral service in the clinic. Women interested to participate in a qualitative follow-up could leave their contact details. Survey questions were pre-tested in a homeless women’s shelter, where one of the authors did voluntary work and therefore knew that a high proportion of women has experienced intimate partner violence, including during pregnancy.

The following two questions were used to assess women’s acceptance of routine or case-based inquiry for intimate partner violence: “Should doctors, according to your opinion, always ask patients during antenatal care about physical or sexual experiences of violence?” and “Should doctors, according to your opinion, always ask non pregnant patients about their physical or sexual violence experiences?” The answer options for both questions were ‘yes’, ‘no’ and ‘under certain conditions’. The later was followed by an open space to provide women with the opportunity to provide further details. If the women answered ‘yes’ they were considered to be in favor of routine inquiry and if they answered ‘under certain conditions’ they were considered to be in favor of case-based inquiry for intimate partner violence. The survey further included questions on the women’s and child’s father’s socio-demographic characteristics and their pregnancy. A modified version of the validated Abuse Assessment Screen (AAS) [26] was used to inquire about psychological, physical and sexual abuse during pregnancy and before pregnancy. Unfortunately the Abuse Assessment Screen has not been validated in Germany yet. To translate the instrument two authors (HS and DS) forward and back translated the AAS.

The qualitative component of the study consisted of in-depth interviews with seven women who experienced violence during pregnancy and who have left their contact details at the end of the survey for follow-up. Women were contacted via email and phone. HS conducted the interviews at the women’s home, after ensuring that this is the most convenient and private place to talk for the women. Interviews were paused if they were interrupted by a third person.

The aim of the qualitative interviews was to support and illuminate the quantitative findings and to explore the conditions under which routine or case-based inquiry for intimate partner violence is acceptable for women in more depth than possible in the quantitative survey. Furthermore, the qualitative interviews explored other suggestions women have on how the health system can assist women who experience intimate partner violence. During the qualitative interviews, women were asked whether their health care provider ever asked them about intimate partner violence. Women who revealed intimate partner violence to their doctor were asked how helpful they felt it was, what could have been done better and what the advantages and disadvantages are of asking all women about abuse. Women who did not reveal intimate partner violence to their doctor, even after being asked about it were probed about the reasons for not revealing violence, the conditions under which they might have revealed violence and what their thoughts are on asking all women about intimate partner violence. All women were also asked on whether they believe antenatal care is a good time for screening and why, or why not.

Ethical approval was granted by the Social Science Division of the University of Oxford and the ethical commission of the medical faculty of the Ludwig-Maximilians-University in Munich.

#### Analysis

Questions on the acceptance of routine or case-based inquiry for intimate partner violence and their association with women’s abuse status were analyzed using chi-square tests and Fisher’s exact tests if the cell count was below five. To assess the association between the acceptance of routine or case-based inquiry for intimate partner violence with women’s socio-demographic factors and adverse health behaviours during pregnancy a multinomial regression analysis controlling for all socio-demographic factors and adverse health behaviours during pregnancy was conducted. Associations with p-values below the 0.05 level were considered significant. The survey was analyzed using STATA 11.

The qualitative interviews were analyzed using a content analysis approach [27]. One author (HS) identified the codes through brief notes made during the transcription of each interview and by re-reading the manuscripts several times. Coding and recoding continued until all the data relating to women’s experiences with routine or case-based inquiry and other health sector interactions were classified [28]. The two authors (HS and DS) discussed the codes until agreement was reached on the codes and the selection of quotations to ensure reliability and to ensure that the data reflects the categories identified.

## Results

### Maternity ward survey

Of the 552 distributed questionnaires, 401 were returned (response rate: 73 percent). The mean age of participants was 33 years (S.D. 5.4, range 18–50), 83 percent were of German nationality, 74 percent were married and 72 percent were in part- or full-time employment before pregnancy.

Of the 401 women, 86 (n = 338, 95% CI: 82.32-89.25) percent were in favour of routine or case-based inquiry for intimate partner violence in general care, with half of the women (51%, 95% CI: 46.06-55.97%, n = 201) supporting routine inquiry, 35 percent (95% CI: 30.04-39.50, n = 137) supporting case-based inquiry and only 14 percent (95% CI: 10.75-17.68, n = 56) opposing both. In antenatal care, acceptance of routine or case-based inquiry was significantly higher with 92 percent (95% CI: 88.90-94.39, n = 362) of women being in favour; 56 percent supporting routine inquiry (95% CI: 51.29-61.12, n = 222), 36 percent (95% CI: 30.71-40.18, n = 140) supporting case-based inquiry and only eight percent (n = 33) opposing both. The corresponding p-value was <0.001 and the chi2 test 392.74.

### Association with women’s experiences of violence

There was no association between women’s acceptance of routine or case-based inquiry for intimate partner violence and their previous experience of violence during antenatal care, regardless of whether they experienced prior violence by a partner or someone else. The exception was women who experienced child sexual abuse. They were significantly less likely to be in favour of routine inquiry for intimate partner violence during antenatal care, but significantly more likely to accept case-based inquiry compared to women who did not report child sexual abuse. Details are displayed in Table 1.

**Table 1:** Influence of experiences of violence on women’s acceptance of routine or case-based inquiry for intimate partner violence in antenatal care

		**Inquiry for intimate partner violence during antenatal care**				
	**Total**	**No**	**Routine**	**Case-based**	**Chi2**	**p-value**
	**N**	**%**	**%**	**%**		
**Physical and/or sexual violence during pregnancy**						
No	386	9	56	36		
Yes	9	0	67	33	0.96	0.619
**Physical or sexual partner violence ever**						
No	373	8	57	35		
Yes	22	9	50	41	0.37	0.831
**Physical or sexual violence ever**						
No	330	8	58	34		
Yes	65	12	45	43	4.59	0.101
**Child sexual abuse**						
No	374	8	58	34		
Yes	21	19	29	52	7.84	0.020

### Associations with socio-demographic and pregnancy-related factors

A multinomial analysis of the demographic and pregnancy related factors showed that women who were in favour of routine inquiry for intimate partner violence in antenatal care were more likely to have completed high school, were aged 22 to 31 years versus being younger than 22 years and did not smoke during pregnancy compared to the women who were against routine or case-based inquiry for intimate partner violence in antenatal care. Women in favour of case-based inquiry for intimate partner violence in antenatal care were more likely to have a high school education, be single or divorced and did not smoke during pregnancy (see Table 2 for details).

**Table 2:** Descriptive statistics and adjusted Odds Rations (95% CI) on the effect of socio-demographic and pregnancy-related factors on women’s acceptance of routine or case-based inquiry for intimate partner violence in antenatal care

		**Acceptance of routine or case-based inquiry**						
	**Total N**	**No**	**Routine inquiry**			**Case-based inquiry**		
		**%**	**%**	**AOR**^**1**^	**CI**^**2**^	**%**	**AOR**	**CI**
Women’s education								
No high school	183	70	45			43		
High school	211	30	55	3.46^*^	[1.00,11.94]	57	3.65^*^	[1.02,13.1]
Women’s employment status								
Not working/seldom	113	27	31			26		
Full- or part-time	282	73	69	0.53	[0.16,1.83]	74	0.53	[0.15,1.90]
Occupational status								
Low	62	27	20			11		
High	300	73	80	2.03	[0.52,7.97]	89	2.86	[0.67,12.2]
Women’s nationality								
German	305	59	77			81		
Not German	62	41	23	0.77	[0.20,2.93]	19	0.67	[0.16,2.75]
Women’s age								
<22	10	11	2			2		
22-31	136	37	39	11.66^*^	[1.16,117.2]	34	6.18	[0.55,68.8]
>31	221	52	59	6.38	[0.65,62.27]	64	3.66	[0.34,39.7]
Child father’s education								
No high school	155	52	42			37		
High school	221	48	58	0.54	[0.16,1.80]	63	0.66	[0.19,2.30]
Child father is employed								
No	32	6	9			8		
Yes	360	94	91	0.40	[0.04,3.67]	92	0.65	[0.06,6.67]
Child father’s nationality								
German	329	79	82			88		
Not German	63	21	18	0.71	[0.18,2.81]	12	0.47	[0.11,2.06]
Women’s marital status								
Married/widowed	292	73	77			70		
Single/divorced	103	27	23	3.60	[0.69,18.76]	30	5.51^*^	[1.02,29.9]
Pregnancy planning								
Planned	300	76	76			76		
Unplanned	52	15	12	1.55	[0.26,9.11]	14	2.17	[0.35,13.4]
Planned later	43	9	12	1.52	[0.33,7.03]	10	1.33	[0.26,6.80]
Keeping antenatal care visits								
No	376	91	95			97		
Yes	19	9	5	0.50	[0.08,3.31]	3	0.18	[0.02,1.83]
Financial difficulties during pregnancy								
No	377	97	97			94		
Yes	16	3	3	1.16	[0.11,12.74]	6	1.82	[0.16,20.6]
Drinking during pregnancy								
No	280	85	70			69		
Yes	115	15	30	3.04	[0.64,14.40]	31	2.94	[0.60,14.3]
Smoking during pregnancy								
No	346	67	89			91		
Yes	49	33	11	0.17^**^	[0.05,0.65]	9	0.14^**^	[0.03,0.59]
Hospital stay during pregnancy								
No	305	70	83			89		
Yes	58	30	17	0.46	[0.14,1.46]	11	0.29	[0.08,1.02]

^*^p < 0.05, ^**^p < 0.01, ^***^p < 0.001; ^1^ AOR = Adjusted Odds Ratio ^2^ CI = 95% Confidence Intervals.

### Open- ended questions

Most of the women (67%, n = 92) who accepted case-based inquiry for intimate partner violence in antenatal care provided further comments on the conditions under which this should be done. Table 3 shows that the majority of women who left a comment suggested that case-based inquiry should only take place if the doctor suspects that the woman experiences intimate partner violence (n = 66). Suspicion may be based on visible signs of abuse, such as injuries, haematoma, the women being scared or fearful or showing deranged behaviour, or knowledge of existing violence or family circumstances. Peculiarities in the behaviour of pregnant women (n = 9) are another reason why doctors should ask women if they experience intimate partner violence. Peculiarities may include physical or psychological abnormalities, such as having unclear medical conditions. A few women also stated that doctors should inquire about intimate partner violence when women ask for an abortion while other wrote that routine inquiry should only take place if women are in the appropriate psychological state for it. Other women stressed that doctors should only ask women about intimate partner violence if they think it is related to the pregnancy and can avoid danger for the mother and the child. Other conditions under which case-based inquiry should take place included requests by individual women to only screen for intimate partner violence if all women will be asked about it, if it is done by female and properly trained doctors and if the women will be informed that their answers are voluntary. Women also noted that doctors should only ask women about intimate partner violence if they feel that the woman would accept help and that the screening has positive consequences.

**Table 3:** Women’s responses to the open survey question on when and under what conditions case-based inquiry for intimate partner violence should take please

	**n**
If the doctor has a suspicion that the woman experiences violence	25
If the doctor observes peculiar behaviour in the pregnant woman	9
To avoid any danger for the child and the mother	2
If women ask for an abortion	2
If the woman raises it herself or wants it to be raised	2
If the doctor can offer the women assistance the woman can choose to take up or not	2
If the question is related to the women’s pregnancy	1
If the mothers is in the right psychological state	1
If the doctor believes that the woman would accept help	1
If all women are asked	1
If a female doctor asks	1
If the doctor has adequate training to ask about violence	1
If the patient is clearly told that her answer is voluntary	1

### In-depth interview

The in-depth interviews of seven women who have suffered from intimate violence are in accordance with the previous findings. All seven women believed that antenatal care is a good time for routine or case-based inquiry for intimate partner violence as women are seeing the same health care provider more often due to the regular antenatal care appointments. This allows them to build up a better relationship and trust with a doctor. They also mentioned women’s desire to secure the health of the unborn baby and doctor’s ability to offer women direct assistance as important criteria for routine or case-based inquiry during pregnancy. At the same time, most of the interviewed women were clear about the importance that doctors should only ask about intimate partner violence if they have sufficient time to raise the question and take women who report abuse serious. They also stressed the importance of privacy, confidentiality, the absence of other persons, and the fact that routine or case-based inquiry for intimate partner violence should have positive consequences. With positive consequences, they meant that the doctor should be able to offer women assistance or referral options if they reveal abuse. The importance of doctors stressing that answers will be treated confidentially seemed especially important for young women who experience intimate partner violence during pregnancy. One of them explained:

“If I really would have been sure that nothing will be forwarded to the public authorities or elsewhere, because there was no danger for the child, .. I think then I would have told him. But the fear [of having the child taken away] was simply too big.”

The interviewed women also voiced their concern that abused women would not admit to the violence by their intimate partners due to shame and fear and the long time they needed themselves before they told anyone of the violence. Although one woman said that she would not have told her doctor about the violence she experienced, she believes that being asked about it might have helped her to realize that the violence was unacceptable and harmful.

There was a discrepancy in women’s answers in the qualitative interviews on whether they preferred a health care provider to ask women directly about violence or to use a more discreet approach. Women who preferred the more discreet approach explained this by the strong need for security they felt during pregnancy, both financially and in regard to preserving an intact family for their unborn child. They rather would have preferred posters and information material in the waiting room that would inform women that their health care provider takes the issue of violence serious and is willing to talk to them about it if they want to. Moreover, a woman under the age of 20 who experienced intimate partner violence during pregnancy suggested increasing the involvement of fathers-to-be in antenatal care, since this would allow the doctor to ask the male partner about how he feels about the pregnancy and the changes it means for the relationship.

## Discussion

Overall, acceptance of routine or case-based inquiry for intimate partner violence during antenatal care was high with 92 percent among women who participated in the maternity ward survey, with 56 percent supporting routine inquiry and 36 percent supporting case-based inquiry. As established previously by a systematic review on the acceptance of routine inquiry for intimate partner violence, our study also found that women are more likely to accept routine or case-based inquiry during antenatal care than general care. These findings support the hypothesis of this study. This may be due to the high frequency of antenatal care visits, the trust that builds up between the health care provider and the woman and women’s desire to have a safe and healthy pregnancy [15].

Acceptance of routine or case-based inquiry was not significantly associated with women’s experiences of violence in adulthood, but with child sexual abuse. Women who experienced child sexual abuse were significantly more in favour of case-based inquiry, but not routine inquiry. Association between the acceptance of routine or case-based inquiry and women’s socio-demographic characteristics and adverse health behaviours during pregnancy were only found with age, education, marital status and smoking during pregnancy. This indicates a need to approach routine or case-based inquiry for intimate partner violence with greater care among younger, less educated and single or divorced women and women who smoke during pregnancy. Literature suggests that some women feel stigmatized if their health care provider inquires about intimate partner violence. This is especially the case if they already feel alienated and offended by discussing substance abuse during pregnancy or mental health problems, which are known to be associated with intimate partner violence [6,24,29,30]. In the qualitative interviews, as well as in previous literature [20,24], it was argued that these women are more afraid of being labelled as inadequate parents and might therefore loose their babies. More research is needed to explore this topic further. The analysis of the open ended questions and the in-depth interviews reveal that routine or case-based inquiry for intimate partner violence needs appropriate training, sensitivity of the doctors, space and time as well as set referral options.

The importance of how a doctor should ask women about intimate partner violence has already been acknowledged in the literature as the complex nature of intimate partner violence results in different situations and needs of women [31,32]. Abused women might need diverse information and assistance, ranging from support from the law enforcement, statutory child protection, criminal, civil and family law to alternative accommodation and therapeutic services and to no services at all. In addition, women’s need of different services may change over time [13].

Women in this study were very clear about the conditions under which routine or case-based inquiry for intimate partner violence should take place, which included sufficient training, time and adequate responses by the health care providers. A recent realist informed systematic review, which investigated how and why routine and case-based inquiry interventions work to inform programmes and policies supports these findings [12]. The review found that programs which had institutional support, effective screening protocols, thorough initial and ongoing training, and immediate access or referrals to onsite and/or offsite support services were most successful in increasing screening and identification rates [12]. Institutional support hereby refers to investment, approval, and support for the integration or institutionalization of the program at higher levels within health care settings or institutions, and occasionally involved making linkages with community resources [12]. This was perceived as crucial to reinforce the necessity of screening, and facilitated support for those who experience intimate partner violence through creating an overall culture of intimate partner violence awareness and its health care-based solutions, and thus seemed to facilitate other program components [12]. The review also found that effective screening protocols were those that promoted screening behaviour and enhanced providers’ perception that they are knowledgeable and equipped to help women who experience intimate partner violence. Effective protocols also clearly outline guidelines on how to ask women about intimate partner violence, how to assess patient safety, review patient options, and refer victims to support services.

The major concern that routine or case-based inquiry for intimate partner violence might be harmful due to women’s disclosure distress or increased perpetration of intimate partner violence if the partner learns that the woman has discussed the abuse with their health care provider [12,33] has been rejected by recent research. A randomized control trial investigating the effectiveness of routine inquiry for intimate partner violence showed that screened women are not reporting harms because of screening [34]. Quite the opposite: Instead of being harmful, women often argue that the screening process itself is the first step in a longer, more complex set of processes that helps them to address the violence in their lives and that reduces their feeling of stigmatization [12,14].

Several limitations of this study have to be considered when interpreting its findings. First, the quantitative survey is cross-sectional and neither the quantitative survey nor the qualitative interviews are based on a representative sample of pregnant women. This significantly limits the generalizability of the findings to the wider population. Second, for ethical and safety reasons, both, the survey and the qualitative interviews were restricted to women who could be met alone or spoke sufficient German. This may have resulted in the exclusion of women who could be at higher risk of experiencing intimate partner violence. Lastly, the survey and the qualitative interviews were based on a small sample of women. Future studies on this issue need to survey a larger, representative sample of women on the acceptability of routine or case-based inquiry for intimate partner violence in antenatal care or other healthcare settings. In addition, there is a need for more qualitative interviews with women who did not experience intimate partner violence on their views on the acceptability of routine or case-based inquiry for intimate partner violence and under which conditions it should be conducted. There is also a need for surveys, focus group discussions and in-depth interviews with health care providers on the acceptability and practicalities of routine and case-based inquiry for intimate partner violence and the conditions under which this would be feasible.

## Conclusion

This study is the first to assess the acceptability of routine or case-based inquiry for intimate partner violence in antenatal care in Germany, using a mixed methods approach. It found that women are highly supportive of routine or case-based inquiry for intimate partner violence in antenatal care, as long as it is conducted by a trained doctor in a professional manner. The study also confirmed that antenatal care indeed is a window of opportunity for doctors to reach women who experience intimate partner violence and to refer them to appropriate assistance. However, more research is needed to establish an acceptable and feasible manner to implement routine or case-based inquiry for intimate partner violence in Germany and to explore German health care providers’ attitudes, perceived barriers and needs to perform routine or case-based inquiry for intimate partner violence. In the meantime, doctors should be encouraged to provide low level interventions, such as disseminating information on available intimate partner violence services in their waiting rooms. Furthermore more awareness needs to be created on the prevalence, health effects and other consequences of intimate partner violence among doctors, nurses and other health care professionals, for example by incorporating it into the training curricular of doctors.

## Competing interest

The authors declare that they have no competing interests.

## Authors’ contributions

HS planned the study, contributed to the data acquisition, performed the data analyses and wrote the first draft of the manuscript. DS contributed to the planning of the study, acquisition of data, interpretation of the data and reviewed and edited the manuscript extensively. LH contributed to the data acquisition and reviewed and edited the manuscript. KF participated in the design of the study and reviewed and edited the manuscript. IH, CB, ND and UH added to the interpretation of the data and reviewed and edited the manuscript. All authors read and approved the final manuscript.

## Pre-publication history

The pre-publication history for this paper can be accessed here:

http://www.biomedcentral.com/1471-2393/13/77/prepub
